# Cognitive and metacognitive factors predict engagement in employment in individuals with first episode psychosis

**DOI:** 10.1016/j.scog.2019.100141

**Published:** 2019-05-01

**Authors:** Abigail C. Wright, Kim T. Mueser, Susan R. McGurk, David Fowler, Kathryn E. Greenwood

**Affiliations:** a University of Sussex, School of Psychology, Brighton, East Sussex, United Kingdom; b Sussex Partnership NHS Foundation Trust, Swandean, West Sussex, United Kingdom; c Center of Excellence for Psychosocial & Systemic Research, Massachusetts General Hospital, USA; d Center for Psychiatric Rehabilitation, Boston University, Boston, MA, USA

## Abstract

**Background:**

Research has demonstrated that cognitive abilities predict work outcomes in people with psychosis. Cognitive Remediation Programs go some way in improving work outcomes, but individuals still experience difficulty maintaining employment. Metacognition has been demonstrated to predict work performance in individuals with schizophrenia, but this has not yet been applied to First Episode Psychosis (FEP). This study assessed whether metacognition, intellectual aptitude and functional capacity can predict engagement in work and number of hours of work within FEP.

**Methods:**

Fifty-two individuals with psychosis, from an Early Intervention in Psychosis service, completed measures of IQ, metacognition (Metacognitive Assessment Interview), functional capacity (UPSA), and functional outcome (hours spent in structured activity per week, including employment).

**Results:**

Twenty-six participants (22 males, 4 females) were employed and twenty-six (22 males, 4 females) were not employed. IQ and metacognition were significantly associated with whether the individual was engaged in employment [IQ (p = .02) and metacognition (p = 006)]. When controlling for IQ, metacognition (differentiation subscale) remained significant (p = .04). Next, including only those employed, no cognitive nor metacognitive factors predicted number of hours in employment.

**Discussion:**

This is the first study to directly assess metacognition as a predictor of work hours for individuals with FEP. This study highlights the importance of enhancing metacognitive ability in order to improve likelihood of, and engagement in, employment for those with FEP.

## 1 Introduction

Employment is central to the concept of recovery in people with severe mental illness (SMI) (McGurk et al., 2009), as finding meaningful roles can be an important part of recovery (Secker et al., 2001; Spaniol et al., 2002). However, employment is generally low in those with schizophrenia (Cella et al., 2016), first episode psychosis (FEP) (Marwaha and Johnson, 2004) and those with an at-risk mental state (ARMS) (Cotter et al., 2017). For example, studies have demonstrated that 15% of those with FEP were employed, although at two-year follow-up this increased to over 45% (Kane et al., 2016), similar to other studies in FEP (Abdel-Baki et al., 2013; Chang et al., 2012; Srihari et al., 2015).

Psychosis has peak onset in late adolescence (Kessler et al., 2007), at the beginning of career-based employment (Killackey et al., 2006), and this early stage of the disorder is typically characterized by impaired academic performance or absences from work or school (Rinaldi et al., 2010; Scott et al., 2013). Occupational outcomes are, therefore, frequently disrupted in persons with FEP (see Rinaldi et al., 2010).

Many people with SMI espouse employment as a long-term recovery goal (Secker et al., 2001) and having paid work is associated with fewer symptoms, better functioning and improved quality of life (Bell et al., 1996; Eklund et al., 2004; Üçok et al., 2012). Whilst some people do engage in meaningful employment, many do not, which has a large social and personal cost (Killackey, 2015; Knapp et al., 2004). Despite advances in psychological interventions for psychosis, occupational outcomes remain poor. There is clear interest in the identification of factors which influence employment in the early stages of psychosis. There are three selected lines of evidence which will be discussed here to suggest factors which predict poor work outcomes and work trajectory: i) neurocognition, ii) functional capacity, iii) interventions (cognitive remediation and supported employment), and iv) metacognition.

Neurocognitive ability has been associated with employment in SMI (Caruana et al., 2018; Evans et al., 2004; Karambelas et al., 2017; McGurk and Mueser, 2004) and, in particular, employment outcomes are shown to be predicted by neurocognitive ability (McGurk and Meltzer, 2000; Metcalfe et al., 2018; Strassnig et al., 2018), independent of psychotic symptoms (Jaeger and Douglas, 1992; Kaneda et al., 2009; McGurk et al., 2018). Functional capacity skills, such as organizing finances, communication skills, and planning activities (Patterson et al., 2001) have been associated with, and considered ecological valid assessments of, neurocognitive ability in schizophrenia and FEP (Vesterager et al., 2012; Bowie et al., 2006, Bowie et al., 2008; Farias et al., 2003). Functional capacity has been consistently associated with functional outcome (Green et al., 2000; Leifker et al., 2009) and, more specifically, employment for those with schizophrenia or bipolar disorder (Mausbach et al., 2010). There is a dynamic interplay between cognitive factors and work outcomes over time (McGurk and Mueser, 2006) and, whilst a global measure of neurocognition can be useful, a more detailed understanding of functional skills may facilitate research efforts to help target care for individuals with FEP.

Evidence for the supported employment model of vocational rehabilitation for persons with SMI has shown promise for both job acquisition and retention (Bond et al., 2012; Drake et al., 2016). It has been suggested that supported employment may work by compensating for the effects of cognitive impairment and symptoms on work (McGurk and Mueser, 2004). Whilst supported employment is beneficial, those with poorer cognitive ability benefit less (McGurk et al., 2018; McGurk et al., 2003).

Additional variables may explain the unaccounted variance in functioning or work outcomes (Green, Kern, & Heaton, 2004; Schmidt et al., 2011). One of these variables may be metacognition (Davies et al., 2017). Metacognition is broadly defined as ‘thinking about thinking’ (Flavell, 1979; Semerari et al., 2003), and has been shown to be poor in individuals with psychosis (Lysaker et al., 2005). Although metacognition is related to neurocognition in psychosis (Davies and Greenwood, 2018), it is considered a distinct factor that is thought to have a crucial role in functioning (Arnon-Ribenfeld et al., 2017), independent of neurocognition and symptoms (Lysaker et al., 2014; Lysaker et al., 2011b). In support of this, studies have shown that metacognitive ability is a mediator between neurocognition, functional capacity and functional outcome in FEP (Davies et al., 2017; Wright et al., 2018a).

Metacognitive ability, measured using the Metacognitive Assessment Scale or the Metacognitive Assessment Interview (MAI) (Semerari et al., 2012), captures the capacity to think about one's own cognitions, emotions and behavior, as well as others', and to use this reflection to respond to challenges (Lysaker et al., 2010a, Lysaker et al., 2010b; Lysaker et al., 2011a). In relation to employment, Lysaker et al. (2010a) demonstrated, within a schizophrenia group, that only those with higher metacognitive ability had higher ratings of work performance over 6 months, even after controlling for neurocognition. There is a need to assess metacognition as a contributor to work outcomes in FEP, as recovery may be more likely earlier on in the course of psychosis (Harrison et al., 2001; Morgan et al., 2014), and engaging in work may facilitate recovery (Provencher et al., 2002). No study has examined whether metacognitive ability is a key correlate of work outcomes in individuals recovering from FEP.

In addition to initial engagement in employment, identifying factors which predict an overall successful job trajectory is important. A successful work trajectory is defined by duration of employment (Teixeira et al., 2018), more hours of work, and more wages earned, which is associated with a moderate increase in life satisfaction (Judge et al., 2010) and less social exclusion (Barry, 1998; Stewart et al., 2009). Identifying factors associated with this can help understand how to improve the duration of employment.

It is thus hypothesized that, for people with FEP, intellectual aptitude (IQ), applied cognitive skills (functional capacity) and metacognition will be associated with likelihood of engagement in employment and, for those in employment, the number of hours worked.

## 2 Methods

### 2.1 Design

This present study involved a cross-sectional design with measures of IQ, metacognition, functional capacity and current employment (using Time-Use survey subscale) in persons receiving services for a recent FEP.

### 2.2 Participants

Individuals with psychosis were recruited through a convenience sample from Early Intervention in Psychosis services in Sussex Partnership NHS Foundation Trust, United Kingdom. Whilst most participants were newly recruited, for some participants this study was at a later time-point as they formed part of a Psychosis Cohort Study (Davies et al., 2017) and a longitudinal study (Wright, Davies, Fowler & Greenwood, under review). There were no differences in rates of employment, hours worked, symptoms, general functioning, metacognition or IQ between those newly recruited into the study compared to those recruited from a previous FEP study.

Inclusion criteria for the study were:

• Enrolled in Early Intervention Services for at least 3 months before entry into the study. All participants received a diagnosis of First Episode Psychosis (Unspecified nonorganic psychosis, F29) by an Early Intervention psychiatrist;
• 18–40 years of age;
• Able to read and communicate in English.

Exclusion criteria for the study were:

• Primary diagnosis of substance use disorder;
• Organic neurological impairment (such as epilepsy, dementias, multiple sclerosis, Parkinson's disease, neuroinfections, brain tumours, and brain trauma)

Data collection was undertaken from March 2017 to April 2018. Participants completed measures of functional capacity, self-reported work hours, IQ, and symptoms. They also completed additional assessments (see Wright et al., 2018b). For the main analyses, we excluded participants who were engaged in childcare (60% of those excluded were female) as this may have prevented them being employed.

## 3 Measures

### 3.1 Basic psychopathology

The Positive and Negative Syndrome Scale (Kay & Fiszbein, 1987) is a widely used instrument assess symptom severity over the past week (Hermes et al., 2012). The Bell et al. (1994) factor structure was used to calculate factor scores for positive (range 6–42), negative (range 8–56), cognitive (range 7–49), hostility (range 4–28), and discomfort (range 4–28) symptom subscales.

### 3.2 IQ

IQ was measured using one test of verbal IQ and one subtest of performance IQ from the WASI-II (Wechsler, 1999). Verbal IQ was measured by the vocabulary task, a measure of an individual's verbal knowledge and fund of information. Performance IQ was measured using the matrix reasoning task, a measure of individual's ability to mentally manipulate abstract symbols and perceive the relationship among them.

### 3.3 Metacognition

Metacognitive ability was assessed using the Metacognitive Assessment Interview (MAI) (Semerari et al., 2012). This requires the participant to reflect on a recent difficult interpersonal experience and to respond to a series of questions. The measure assesses the individual's ability on four subscales: i) monitoring: identification of feelings and thoughts; ii) differentiation: distinguishing between dreams, beliefs or assumptions; iii) integration: reflecting on different mental states and rules governing them; and iv) decentralization: describing the mental state of the other person which is independent of their own view. The interview was transcribed and scored on the four subscales between 0 (skill is not evident) to 5 (sophisticated), depending on spontaneity, use of aids/prompts and the sophistication of the answers. The scores for the sub-domains (monitoring, differentiation, integration and decentralization) were averaged to provide a total composite score. This measure has demonstrated good inter-rater reliability and internal consistency =0.90 for total metacognition), factorial validity, and reliability (r = 0.62 to 0.90) (Semerari et al., 2012).

### 3.4 Function

#### 3.4.1 Employment

Employment was assessed using the Time-Use Survey (TUS) (Short, 2006). TUS is a structured interview (inter-rater reliability 0.99; Hodgekins et al., 2015) during which participants are asked questions regarding the number of hours spent engaged in specific structured activities for the preceding month (Fowler et al., 2009), including hours spent in paid work, voluntary work, educational activity, childcare, sports, leisure and housework activities. A weekly average was calculated for each activity. This measure is able to capture differences across clinical groups (Cella et al., 2016; Hodgekins et al., 2015). The present study focused on hours of paid employment and also a binary variable was created for paid work in the past week: 0 (no work) or 1 (any work).

#### 3.4.2 Functional capacity

The UCSD Performance-Based Skills Assessment (Patterson et al., 2001) is a role play test designed to assess the capacity to perform a range of basic functional and social behaviors based on a series of simulated tasks. This measure assesses performance skills in five areas, including: i) finances, ii) communication, iii) comprehension/planning, iv) use of transportation, and v) household activities. The participant is provided with equipment and asked to complete a list of tasks designed to replicate skills required in everyday life. During each role-play the individual was given 0 or 1 point(s), following manual guidelines. These raw scores were totaled for each domain and converted into 0–10 scale to be comparable across domains. This score was then multiplied by 2 and summed to provide a total out of 100. This measure demonstrates high internal consistency (α = 0.88), good validity with other scales (Direct Assessment of Functional Status scale, r = 0.86) and good test-retest reliability (r = 0.91) (Harvey et al., 2007; Mausbach et al., 2011).

### 3.5 Statistical analysis

Using the binary work variable (within employment in the last month or not), individual logistic regressions were conducted to assess whether the composite scores for IQ, functional capacity or metacognitive ability predicted the likelihood of the participant being employed. Next, using the most significant sub-domain of the significant predictors (e.g. metacognition), a multiple logistic regression was conducted to assess unique predictors of likelihood of employment. Similar linear regression analyses were conducted among the participants who worked to evaluate predictors of number of hours worked, with IQ, functional capacity, and metacognition as individual predictors.

## 4 Results

### 4.1 Sample characteristics

A total of fifty-two participants (81% male) completed the study. The mean age was 26.13 years(SD = 5.5, range 18–43) (see Table 1).

**Table 1:** Sample characteristics summary table.

	N = 52	%
Gender		
Male	42	81%
Female	10	19%
Antipsychotic medication^a^		
Yes	36	69%
No	16	31%
Education (level, %)		
No qualifications	2	4%
GCSE	15	29%
A-levels	19	36%
Degree	14	27%
Higher degree	1	2%
Prefer not to say	1	2%
Work status		
Full-time	16	31%
Part-time	7	14%
Not in employment	12	23%
Student	9	17%
Looking after family/home	1	2%
Long-term sickness	6	11%
Other	1	2%
Accommodation status		
Parent's house	21	40%
Other family home	2	4%
Lives independently	23	44%
Supported living	2	4%
NHS accommodation	1	2%
Other	2	4%
Prefer not to disclose	1	2%

a Self-reported and confirmed with clinical records.

### 4.2 Descriptive statistics

All predictor and outcome variables were checked for skewness, kurtosis and outliers. (See Table 2).

**Table 2:** Descriptive statistics for the measures in FEP participants: means (standard deviations).

N = 52	Mean (S.D)	In employment Mean (S.D)	Not in employment Mean (SD)	Difference test
Age (years)	26.13 (5.5)	26.6 (0.5.94)	25.6 (5.1)	t(50) = 0.68, p = .5
Vocabulary task (t-score)	52.3 (13.39)	56.0 (11.7)	48.9 (14.1)	t(48) = 1.9, p = .06
Matrix Reasoning task (t-score)	52.73 (8.59)	53.7 (8.3)	51.8 (8.9)	t(49) = 0.81, p = .42
2-part IQ	105.9 (15.19)	111 (12.3)	101.4 (16.3)	**t(47)** **=** **2.3, p** **=** **.03**
Metacognitive Assessment Interview (MAI) total ^0–5^	3.19 (0.91)	3.52 (0.65)	2.86 (1.0)	**t(42.5)** **=** **2.83, p** **=** **.01**
MAI Monitoring^0–5^	3.49 (0.85)	3.7 (0.74)	3.2 (0.88)	**t(50)** **=** **2.2, p** **=** **.04**
MAI Differentiation^0–5^	2.87 (1.15)	3.3 (0.83)	2.4 (1.3)	**t(43.3)** **=** **3, p** **=** **.01**
MAI Integration^0–5^	3.13 (0.99)	3.5 (0.75)	2.8 (1.1)	**t(43.9)** **=** **2.4, p** **=** **.02**
MAI Decentralism^0–5^	3.25 (1.01)	3.6 (0.78)	2.9 (1.1)	**t(44.5)** **=** **2.4, p** **=** **.02**
UPSA Finance ^0–20^	17.31 (2.68)	17.8 (2)	16.8 (3.2)	t(39.8) = 1.4, p = .18
UPSA Communication^0–20^	13.59 (3.27)	14.5 (2.6)	12.6 (3.6)	**t(48)** **=** **2.1, p** **=** **.04**
UPSA Comprehension and planning^0–20^	14.62 (3.71)	15.6 (3.1)	13.6 (4.1)	t(48) = 1.9, p = .06
UPSA Transport^0–20^	16.93 (3.23)	16.8 (3.7)	13.6 (4.1)	t(48) = −0.29, p = .78
UPSA Household^0–20^	17.9 (3.05)	18.2 (3.2)	17.6 (2.9)	t(48) = 0.69, p = .49
UPSA Total ^0–100^	77.24 (18.56)	82.9 (9.1)	77.8 (10.9)	t(48) = 1.8, =0.08
Time-Use Total (hours in structured activity)	33.32 (19.0)	45.7 (15.0)	20.9 (13.9)	**t(50)** **=** **6.2, p** **<** **.001**
Time-Use Employment (hours per week)	25.0 (12.16)			
PANSS Positive ^(6–42)^	11.46 (4.71)	9.7 (3.1)	13.2 (5.4)	**t(38.8)** **= −2.9, p** **=** **.01**
PANSS Negative ^(8–56)^	13.69 (5.82)	12.4 (2.7)	15.0 (7.6)	t(29.7) = −1.6, p = .11
PANSS Cognitive ^(7–49)^	11.14 (3.42)	10.0 (2.0)	12.2 (4.2)	**t(34.8)** **= −2.4, p** **=** **.02**
PANSS Hostility ^(4–28)^	5.48 (1.54)	5.5 (1.2)	5.4 (1.8)	t(48) = 0.18, p = .86
PANSS Discomfort ^(4–28)^	9.74 (3.72)	8.8 (3.3)	10.7 (3.9)	t(48) = −1.83, p = .07

*Note*: MAI = Metacognitive Assessment Interview; UPSA = UCSD Performance-Based Skills Assessment; PANSS = Positive And Negative Syndrome Scale. **Bold** = significant differences.

### 4.3 Hypothesis 1

Among the 52 participants, 26 (22 males, 4 females) had worked over the past month and 26 (22 males and 4 females) had not worked. The logistic regression analyses indicated that IQ (Ӽ^2^ = 5.32, df = 1, p = .021) and metacognitive ability (Ӽ^2^ = 7.5, df = 1, p = .006) predicted employment status, whereas functional capacity (UPSA) did not (p = .07). See Table 2 for means for those employed or not employed. IQ explained 13.7% (Nagelkerke R2) of the variance in likelihood of employment and correctly classified 59% of the cases. Metacognitive ability explained 17.9% (Nagelkerke R2) of the variance in likelihood of employment and correctly classified 65% of the cases.

The logistic regressions on the two individual tests of the IQ measure indicated a marginally significant effect for vocabulary score (Ӽ^2^ = 3.75, df = 1, p = .053) and a non-significant effect on matrix reasoning (p = .41). All the logistic regressions on the MAI subscales were significant: monitoring (Ӽ^2^ = 4.61, df = 1, p = .032), differentiation (Ӽ^2^ = 8.21, df = 1, p = .004), integration (Ӽ^2^ = 5.67, df = 1, p = .017) and decentralism (Ӽ^2^ = 5.62, df = 1, p = .018).

When including both IQ and MAI (differentiation subscale) were included in the analysis, the full logistic regression model was statistically significant (Ӽ^2^ = 9.4, df = 1, p = .009). The odds ratio for metacognition is 1.9 [CI 1, 3.5] with a large Beta value (0.63), in comparison to IQ with odds ratio 1.03 [CI 0.98, 1.08] with a small Beta value at (0.03), demonstrating metacognition has the largest effect on likelihood of employment. Due to the correlations between IQ and MAI differentiation [r = 0.51, p < .001], neither IQ nor metacognition were significant in the model (Table 3). Multicollinearity assessments were within reasonable limits.

**Table 3:** Multiple logistic regression to predict likelihood of employment.

		B	SE B	Wald	p value	95% CI
Step 1						
	IQ	0.03	0.03	1.02	0.31	0.98, 1.08
	MAI - differentiation	0.63	0.32	3.79	0.052	1, 3.5
	Constant	−4.67	2.5	3.49	0.062	

### 4.4 Hypothesis 2

Next, including only those who worked within the last month (N = 26), logistic regression analyses were conducted to assess whether the composite scores for IQ, functional capacity or metacognitive ability predicted number of hours in work. Mean hours in employment = 25.0 (SD = 12.16, range 2.71–42 h per week). Neither IQ, MAI total, nor UPSA, predicted number of hours worked (p > .05).

## 5 Discussion

Within this study, half of those with FEP were employed (50%), which is similar to others (Abdel-Baki et al., 2013; Srihari et al., 2015) and close to Kane et al. (2016) at 2 year follow-up. This was the first study to demonstrate that, for those with FEP, higher IQ and more efficient metacognitive ability was associated with likelihood of employment. The demonstrated role of IQ on work in this study is consistent with literature in SMI showing that neurocognitive ability predicts work outcomes (McGurk and Mueser, 2004; Metcalfe et al., 2018). Further, results showed that the relationship between IQ and employment is present early in the illness. This study did not demonstrate the role of functional capacity skills on engagement in employment, inconsistent with previous research in schizophrenia (see Mausbach et al., 2010), suggesting this relationship may be different in FEP. Given that the UPSA is highly associated with cognitive functioning (Bowie et al., 2008), the lack of association between UPSA and work may indicate that, whilst cognitions are important, real-life functional skills are less important to engagement in work in this FEP sample. Instead, the core cognitive skills have a key role, following previous research (see (Karambelas et al., 2017; Nuechterlein et al., 2011).

Metacognitive ability was associated with whether individuals with FEP were employed. This suggests that an individual's ability to think about their thoughts, feelings and experiences in a sophisticated, yet flexible, manner predicted whether the individual was able to obtain and maintain employment. This supports previous research in schizophrenia (Lysaker et al., 2010a). Metacognitive ability also entails one's ability to use strategies to deal with difficult situations in the workplace as they arise and reflect ones' awareness of social roles and relationships with others. Therefore, compromised metacognition may interfere with the ability to appreciate the unwritten rules of work place behavior, such as what constitutes personal space, specific boundaries within the work environment, or rules of social disclosure. A lack of appreciation of such rules can be costly in terms of job retention.

Importantly, the effect of metacognition on engagement in employment was stronger than the effect of IQ. In particular, the metacognitive subscale “differentiation” was strongly associated with likelihood of employment in this FEP sample. Metacognitive differentiation concerns the ability to consider one's own opinion as a hypothesis (Semerari et al., 2012). Those who experience difficulties in recognizing the fallibility of their own thinking may not be able to correct their own inaccurate interpretations of other's intentions and, as a result, may experience difficulty working alongside others and maintaining employment. These results suggests the importance of training both cognitive and metacognitive factors to maintain employment in FEP.

Despite the association between both IQ, metacognition and likelihood of engagement in employment, these factors did not predict engagement in working more hours. This may be a self-report issue related to errors in reporting work amounts or a power issue. Future studies could aim to recruit a larger sample to assess all individual predictors within an adequately powered model. Alternatively, it may be suggested that both cognitive and metacognitive ability must be above a certain threshold to enable the individual to gain employment or additional, untested, factors may improve the amount of employment (for example, job satisfaction, motivation, or physical health problems).

In terms of clinical implications, lower IQ and lower metacognitive ability may be markers for those with FEP who will experience difficulties in obtaining employment. These individuals may require assistance in gaining employment, using combined cognitive remediation and vocational rehabilitation (e.g. the Thinking Skills for Work program; McGurk et al., 2005) or by training metacognitive ability, such as Metacognitive Insight and Reflection Therapy (MERIT), which is aimed at helping individuals integrate their experiences and improve their ability to manage difficulties (Lysaker and Klion, 2017). Currently, Early Intervention Services within the UK offer employment or education support and, whilst this study did not assess the use of these services, this study has provided evidence to suggest the potential usefulness of integrating metacognitive training within current supported employment services. The findings highlight the clear importance in addressing cognitive and metacognitive difficulties before engaging the individual in employment.

## 6 Limitations

Firstly, the self-report nature of work may not be as accurate as other forms of determining work status and amounts. Secondly, we had to exclude individuals who were engaged in childcare responsibilities as this reduced the opportunity to engage in employment. This excluded a number of females who were performing higher on functioning, metacognition and cognition than males and, in turn, also reduced the sample size and variance. Thirdly, individuals engaged within this research study typically scored higher on functioning and cognitive measures and lower on symptom scores than previous studies of people with FEP (Hodgekins et al., 2015; Leucht et al., 2005) and a larger number were engaged in work than previous studies (Karambelas et al., 2017). This group may be higher functioning than the average FEP group and less representative of those with poorer outcomes. As this current FEP sample were performing higher on these factors than a typical FEP sample, this may have reduced the variance in the scores and therefore would have required a large sample of participants. Fourthly, substance use has been previously associated with employment outcomes in SMI (McGurk et al., 2009; Richardson & Epp, 2016) and whilst this study excluded individuals who had a diagnosis of substance misuse disorder, use of substances in SMI is high (Dharmawardene & Menkes, 2015; RachBeisel, Scott & Dixon, 1999) and under-reported (Bahorik et al., 2014). Future research should aim to replicate this study whilst using more rigorous controls for substance use. Finally, other aspects of metacognition could also be explored, such as metacognitive experience (a form of self-knowledge where one can attribute beliefs to performance), previously associated with real-world functioning (Gould et al., 2015), which may provide insight into additional predictors of employment.

## 7 Conclusion

This study was the first study to demonstrate the combined roles of IQ and metacognition on predicting likelihood of employment. However, for those within employment, none of the variables assessed predicted the number of hours the individual was engaged in work. This suggests different factors predict whether someone was motivated to engage in employment and whether they were motivated to engage in more hours of employment. Employment programs should consider clients' cognitive and metacognitive skills to help inform essential services to compensate for difficulties in these areas, as well as to engage the individual within employment and maintain employment overtime, to improve mental health recovery.

## Funding statement

This work was supported by Sussex Partnership NHS Foundation Trust, United Kingdom and Economic Social Research Council, United Kingdom, through a PhD studentship awarded to the first author (Reference: ES/J500173/1).

Ethical and Health Research Authority approval was obtained through Camberwell St. Giles Research Ethics Committee (reference number: 17/LO/0055).
